# Current perspectives on mesenchymal stromal cell therapy for graft versus host disease

**DOI:** 10.1038/s41423-023-01022-z

**Published:** 2023-05-10

**Authors:** Nadir Kadri, Sylvie Amu, Ellen Iacobaeus, Erik Boberg, Katarina Le Blanc

**Affiliations:** 1 Department of Laboratory Medicine, Karolinska Institutet, Stockholm, Sweden; 2 Department of Clinical Neuroscience, Division of Neurology, Karolinska Institute and Karolinska University Hospital, Stockholm, Sweden; 3 Department of Haematology, Karolinska University Hospital, Stockholm, Sweden; 4 Department of Cell Therapies and Allogeneic Stem Cell Transplantation Karolinska University Hospital, Stockholm, Sweden

**Keywords:** MSC, GVHD, Hematological malignancies, acute GVHD, chronic GVHD, Mechanisms of disease, Cancer

## Abstract

Graft versus host disease (GvHD) is the clinical condition in which bone marrow-derived mesenchymal stromal cells (MSCs) have been most frequently studied. In this review, we summarize the experience from clinical trials that have paved the way to translation. While MSC-based therapy has shown an exceptional safety profile, identifying potency assays and disease biomarkers that reliably predict the capacity of a specific MSC batch to alleviate GvHD has been difficult. As GvHD diagnosis and staging are based solely on clinical criteria, individual patients recruited in the same clinical trial may have vastly different underlying biology, obscuring trial outcomes and making it difficult to determine the benefit of MSCs in subgroups of patients. An accumulating body of evidence indicates the importance of considering not only the cell product but also patient-specific biomarkers and/or immune characteristics in determining MSC responsiveness. A mode of action where intravascular MSC destruction is followed by monocyte-efferocytosis-mediated skewing of the immune repertoire in a permissive inflammatory environment would both explain why cell engraftment is irrelevant for MSC efficacy and stress the importance of biologic differences between responding and nonresponding patients. We recommend a combined analysis of clinical outcomes and both biomarkers of disease activity and MSC potency assays to identify patients with GvHD who are likely to benefit from MSC therapy.

## Introduction

Mesenchymal stromal cells (MSCs) were initially recognized for their ability to support hematopoiesis and trilineage differentiation capacity [1]. To date, MSCs have been identified in nearly all tissues and provide both structural and trophic support to neighboring organ-specific cells [2, 3]. In addition, MSCs possess potent immunomodulatory effects that influence both adaptive and innate immune cells. The paracrine effects of MSCs are not fixed and rather occur in response to their microenvironment. When surrounded by activated immune cells, MSCs exert potent anti-inflammatory effects.

Although many trials have been performed, unequivocal support of a significant anti-inflammatory effect in a clinical context has been difficult to obtain. The most commonly studied outcome is a reduction in unfavorable immune reactions after hematopoietic stem cell transplantation (HSCT), so-called graft versus host disease (GvHD). However, the road to translation of MSC-based therapy for GvHD treatment has proven to be long and complicated.

Patient characteristics likely influenced the clinical outcomes of individual patients, and species differences confounded early attempts to decipher the MSC mode of action (MoA) in experimental animal models of GvHD. Instead, encouraging results obtained from small clinical trials in combination with biological in vitro and in vivo investigations of MSC donor and recipient responses paved the way for hypotheses of the MSC MoA after adoptive transfer. Experimental animal models corroborated the clinical findings, leading to our current understanding of how MSCs alleviate GvHD. Finally, in 2022, the first randomized trial indicated that MSCs not only had a beneficial effect in acute GvHD (aGvHD) but also reduced the risk of subsequent development of chronic GvHD (cGvHD) [4].

In this review, we summarize preclinical and clinical studies that have led to the introduction of MSCs into the treatment arsenal for acute and chronic GvHD. We discuss the role of disease biomarkers and the interactions between MSCs and immune cells associated with GvHD resolution, as well as mechanistic findings made using murine GvHD models.

## Hematopoietic stem cell transplantation

For patients with high-risk leukemia, allogeneic HSCT is the only curative regimen. Engrafted donor-derived lymphocytes provide continuous surveillance and eliminate residual leukemic cells, a phenomenon termed the graft-versus-leukemia (GvL) effect. However, donor lymphocytes may also attack the recipient’s healthy tissue, resulting in a clinical manifestation termed GvHD (as reviewed by Socié and Ritz [5]). Several factors influence the risk of developing GvHD, including donor and recipient age and sex mismatch, human leukocyte antigen (HLA) disparity, conditioning regimen toxicity and the source of the hematopoietic stem cell graft. Half of transplanted patients go on to develop GvHD, making it a major factor preventing successful treatment.

GvHD is classified as either acute or chronic based on distinct clinical presentations. Timing is also important, as aGvHD usually occurs within 100 days posttransplantation, while cGvHD onset occurs later [6]. Along with infections and, to a lesser extent, secondary neoplasia, GvHD is considered a main cause of nonrelapse mortality (NRM).

Acute GvHD is thought to be initiated by treatment-related tissue damage and the release of proinflammatory cytokines, which activate donor T cells that attack recipient major and minor histocompatibility antigens. Once activated, donor T cells migrate to target organs and stimulate the recruitment of other effector cells, such as cytotoxic T cells and natural killer (NK) cells. These effector cells cause further damage through direct cytotoxicity or by cytokine release, propagating the inflammatory response. The skin, gastrointestinal (GI) tract and liver are the most commonly affected organs [5]. Disease severity at onset and the extent of organ involvement can be used to categorize aGvHD into four subtypes that predict both survival and response to therapy: I (mild), II (moderate), III (severe), and IV (very severe) [7, 8].

Most cases of cGvHD are diagnosed during the first year after HSCT, with cGvHD affecting 35–50% of patients [9, 10]. The initiating events are thought to be the same as those for aGvHD, but cGvHD is characterized by prolonged inflammation with loss of central and peripheral tolerance resulting in the dysregulation of T cells and B cells and a deficiency of regulatory subsets. Activated matrix-producing myofibroblasts, stimulated by cytokines such as platelet-derived growth factor α (PDGFα) and transforming growth factor β (TGFβ), cause fibrosis that can affect most tissues and organs. Our poor understanding of cGvHD etiology has limited the development of targeted treatments. To date, biomarkers for predicting response to treatment and prognosis are all lacking, but the proinflammatory chemokines CXC-chemokine ligand 9 (CXCL9), CXCL10 and B-cell activating factor (BAFF) have been reported to be increased in patients with cGvHD [11].

Elevated CXCL9 at day +100 was recently reported in patients who later developed severe cGvHD, suggesting its potential use as a predictive marker. Another study reported elevated levels of CXCL10 in patients with cGvHD of short duration compared to patients with longstanding disease [12]. Markers that can distinguish patients with active cGvHD who need immunosuppressive therapy from patients with cumulative organ damage but no inflammation are important for optimal patient care. A differential metabolomic profile in cGvHD patients may also provide a signature indicative of active disease [13, 14].

The primary treatment for significant acute and chronic GvHD is systemic corticosteroids, which provide improvement in most patients [15]. Steroids have a wide array of anti-inflammatory effects, inducing T-cell apoptosis and suppressing macrophage activation and cytokine release. Calcineurin inhibitors are commonly added to the treatment for their steroid-sparing effects.

However, patients with visceral and/or multiorgan involvement are generally less responsive to steroids [8]. Acute GvHD that is unresponsive to 3–14 days of steroid treatment is defined as steroid-refractory (SR-aGvHD) [16]. Persistent nonresponsiveness to primary therapy after four weeks is associated with long-term NRM ranging between 40% and 70% [17].

The degree of clinical response following four weeks of systemic steroid therapy is predictive of long-term outcome and has therefore been a relevant primary endpoint in aGvHD treatment trials. Patients with full clinical improvement are usually classified as those with complete response (CR), while patients with incomplete improvement are classified as those with partial response (PR). The term overall response (OR) refers to the sum of the PR and CR rates.

While the classification of aGvHD severity is traditionally based on clinical symptoms, biological biomarkers have the potential to both accurately identify patients with high-risk disease and serve as indicators of responsiveness to a specific treatment. The Mount Sinai Acute GvHD International Consortium (MAGIC) has identified a prognostic algorithm based on two serum biomarkers, regenerating islet-derived 3α (REG3α) and suppressor of tumorigenesis 2 (ST2). Both reflect the extent of crypt damage in the GI tract and are superior predictors of long-term outcome compared to clinical symptoms [18]. Patients with persistent lower GI GvHD exhibit poor prognosis with an overall survival (OS) rate of 25% at 2 years [19], hence the importance of biomarkers to identify this particular group of patients.

The clinical presentation of cGvHD resembles that of autoimmune disorders. The NIH Chronic GvHD Consensus Conference papers have established and refined standard definitions for cGvHD diagnosis and response to therapy, involving evaluation of the many organs and tissues that can be affected [20, 21]. The more recent 2020 Chronic GvHD Consensus Conference publication stressed the importance of early recognition of symptoms as well as the need for the identification of biomarkers predictive of the development of therapy-resistant cGvHD [22].

A definite consensus regarding the optimal second-line therapy for GvHD is still lacking, but several drugs have received regulatory approval in the last few years [23]. Pharmacotherapies such as inolimomab (an anti-CD25 antibody), ABX-CBL (a hybridoma-generated murine IgM monoclonal antibody against the CD147 antigen), and anti-thymocyte globulin (ATG) have all failed to show superiority in randomized trials of aGvHD [24–26]. Patients with cGvHD that are either refractory or dependent on steroids may be treated with a variety of second-line treatments, including photopheresis and ibrutinib [27, 28]. Based on a randomized phase II study, belumosudil, an oral selective ROCK2 inhibitor regulating Th17/regulatory T-cell balance, was approved by the FDA in 2021 for patients with cGvHD who received at least 2 prior lines of treatment [29].

Ruxolitinib was recently demonstrated to induce higher OR and failure-free survival in recent studies of both steroid-refractory acute and chronic GvHD when compared to best available care defined as the investigator’s choice of therapy [30–32]. The overall response of those with aGvHD was 54.9% at Day 28 in the phase II trial and 62% in the randomized trial, with a durable OR of 40% at Day 56. Following the results from these trials, both the FDA and EMEA have over the last two years approved ruxolitinib as a treatment for both SR-aGvHD and cGvHD. However, that there has been discontinuation of treatment due to side effects, including cytopenias, infections and lack of response, indicates the need for additional therapies.

## First wave-the first clinical msc reports and approval in Japan

Culture-expanded autologous MSCs were first infused in humans in a safety trial [33]. Subsequent trials aimed to promote hematopoietic recovery in patients undergoing myeloablative therapy for breast cancer. However, MSCs from patients recently treated with chemotherapy grow poorly in vitro, limiting the clinical use of autologous MSC therapy in cancer patients [34].

Following in vitro studies that demonstrated strong immunomodulatory properties of MSCs, it was postulated that adoptive transfer of allogeneic MSCs may be applied to suppress disease activity in SR-aGvHD. The clinical outcome of the first patient treated, reported in 2004, showed that infusion of haploidentical MSCs from the patient’s mother improved GI and hepatic manifestations of severe GvHD [35]. Withdrawal of calcineurin inhibitor therapy led to reoccurrence of GvHD symptoms, but a second MSC infusion improved GvHD in the patient once again. Encouraged by this proof-of-principle case, eight additional patients with SR-aGvHD were treated at our center, six of whom had favorable treatment responses [36].

The clinical benefit of MSC infusion was further corroborated in a European collaborative, nonrandomized phase II trial using a shared ex vivo cell expansion protocol [37]. In this trial, 55 patients, including 30 adults and 25 children with severe SR-aGvHD, were treated with allogeneic HLA-identical, haplo-identical, or mismatched MSCs. The study patients were all severely ill, mainly suffering from GvHD of the GI tract and liver. Twenty-seven patients received a single MSC infusion, and the remaining patients were treated with two or more infusions. Thirty patients achieved CR, and nine patients achieved partial clinical improvement. No side effects were observed during or immediately after MSC infusion. When comparing patients with CR to nonresponders, decreased transplantation-related mortality (TRM) one year after infusion (37% vs. 72%; *p* = 0.002) and increased OS two years after HSCT (53% vs. 16%; *p* = 0.018) were observed. In the following years, the results of the European collaborative study were confirmed by multiple studies performed around the world [38–46]. One study used MSCs as first-line treatment in association with steroids, while the other studies only included patients with failure of one or several medications [47].

The early studies were reassuring regarding several concerns. They unanimously reported a consistently high safety profile associated with MSC infusion. In the first extensive, systematic review and meta-analysis in 2012, Lalu et al. summarized the safety of systemic MSC administration [48]. The findings were further confirmed by the same group in 2020 [49] and later also corroborated by Ying Li et al. [50]. There is no evidence that MSC adoptive transfer causes severe infusion-related toxicity, organ system complications, infections, death, or malignancy [48, 49]. MSCs do not appear to increase the risk of leukemic relapse or themselves undergo malignant transformation in the recipient [51]. An early study reported that patients were at continued risk of infectious complications several years after MSC infusion and resolution of aGvHD [52]. However, oral antifungal and antiviral prophylaxis were not available in previous decades. Today, it is widely acknowledged that patients with severe GvHD remain immunocompromised as a result of both the disease itself and immunosuppressive treatment regimens and therefore require antifungal and antiviral prophylaxis as part of routine clinical management.

Although the initial clinical studies were not powered to assess efficacy, the results were encouraging and suggested increased survival in CR patients. Chen et al. conducted a careful meta-analysis of thirteen studies including 301 MSC-treated patients with SR-GvHD [53]. Response occurred in 205 patients. Patients with GvHD of the skin had higher response rates than patients with GI manifestations (CR: odds ratio=1.93, 95% confidence interval [95% CI]: 1.05–3.57, *p* < 0.05) or liver manifestations (CR: odds ratio=2.30, 95% CI: 1.12–4.69, *p* < 0.05, and odds ratio=2.93, 95% CI: 1.06–8.08, *p* < 0.05). Furthermore, MSC recipients with grade II disease had better clinical responses than patients with grade III–IV GvHD (CR: odds ratio=3.22, 95% CI: 1.24–8.34, *p* < 0.05).

The clinical MSC product has varied between different studies; both HLA-matched, haploidentical cells and mismatched cells have been used. Interestingly, clinical responses do not appear to be influenced by cell culture conditions, including the use of fetal bovine serum (FBS) or human platelet lysate in the culture medium, or by the degree of HLA disparity or ABO matching between MSC donors and recipients [54].

In contrast to the promising results reported in earlier phase trials, a large multicenter phase III clinical trial conducted in the USA between 2006 and 2009 assessing the use of an industrial MSC product (remestemcel-L, Prochymal) failed to meet its primary clinical endpoint, defined as complete resolution of aGvHD symptoms for at least 28 days after beginning the treatment [55]. Over the course of four weeks, 260 patients ranging in age from six months to 70 years were randomly assigned in a 2:1 manner to receive eight intravenous (i.v.) infusions of remestemcel-L or placebo. Remestemcel-L proved to be safe and well tolerated. Per institutional guidelines, additional second-line therapies were administered. The negative outcome of the study left the MSC field confronted with a paradox regarding the clinical utility of MSCs for GvHD.

The study included patients with skin, liver and GI GvHD, and response in the MSC-treated group as a whole was not statistically superior to that of the placebo arm. However, post hoc analyses of patients with liver involvement revealed both higher CR and PR rates in the remestemcel-L group (29% compared to 5% in placebo patients; *p* = 0.047). The results were similar when patients with high-risk disease were analyzed separately; remestemcel-L demonstrated a significantly higher OR at Day 28 than placebo (58% versus 37%; *p* = 0.03).

There was also a trend toward a superior clinical response in children compared to adult patients, an observation that agreed with the findings presented by Le Blanc et al. [36]. Favorable responses in pediatric patients were also reported by Ball et al., who detailed the results of a retrospective analysis of a cohort of 37 children aged 3 months to 17 years suffering from grade III and IV SR-GvHD treated with allogeneic MSCs [44]. Patients with CR after MSC therapy had a cumulative incidence of TRM of 17% compared to the 69% TRM of patients who were unresponsive to MSCs (*p* = 0.001). Overall survival was 37% after a median follow-up period of 2.9 years (65% in CR patients and 0% in non-CR patients; *p* = 0.001). It remains unknown whether the higher efficacy of MSC treatment in pediatric patients is due to inherent properties of the MSCs, age-dependent variations in alloreactivity or both [56].

A separate study of 75 children with severe aGvHD failing first-line treatment and, for the most part, second-line treatment was reported just one year later by Kurtzberg and colleagues [57]. Patients received biweekly infusions of 2 million MSCs/kg for four weeks, consistent with the schedule of the previous remestemcel-L trial. Patients with either PR or mixed response on Day 28 were eligible for an additional weekly MSC infusion for four more weeks. On Day 28, the overall response was 61.3%. On Day 100 following MSC infusion, the clinical response correlated with significantly improved survival. Compared to nonresponding patients, patients who responded to MSC treatment by Day 28 had a significantly higher Kaplan‒Meier-estimated probability of surviving to Day 100 (78.1% versus 31.0%; *p* < 0.001).

In 2003, Japan Credit Rating Co., Ltd. obtained orphan designation and license from Osiris Therapeutics Inc. to manufacture the third-party MSC product JR-031, similar to remestemcel-L. In the first multicenter phase I/II study, 14 patients (one child and 13 adults) suffering from grade II (*n* = 9) or III (*n* = 5) SR-aGvHD were treated with allogeneic MSCs according to the same schedule used in the trials with remestemcel-L, with no additional second-line agents given. By week four, 13 of 14 patients (92.9%) responded to MSC therapy with CR (*n* = 8) or PR (*n* = 5) [58].

Another 25 patients were treated with MSCs (JR-031) according to the same dosing schedule in a follow-up phase II/III trial. Steroid refractory aGvHD was defined as disease progression after three days or stable disease after five days of corticosteroid treatment. During MSC treatment, no additional immunosuppression was given [58]. The primary endpoint, durable CR by 24 weeks, was obtained in 12 of 25 patients (48%). Twelve patients treated with either MSCs as a single agent (6 patients) or MSCs followed by subsequent third-line therapy (6 patients) were alive with CR at 52 weeks.

In 2015, the Japanese Pharmaceuticals and Medical Devices Agency granted approval to JR-031 (TEMCELL®) for the treatment of aGvHD in both children and adults based on the findings of these studies.

## Second wave–more studies and more confusion

Protocols for ex vivo MSC expansion vary between academic centers [59, 60]. To avoid the risk of zoonotic infections, human platelet lysate has largely replaced fetal bovine serum as the cell culture supplement. Time in culture may affect cell characteristics, and early-passage cells have been suggested to be more potent than batches of extensively expanded cells, perhaps indicating differing degrees of cell senescence [61]. MSCs from different donors vary in their ability to expand and differentiate [62]. In addition, whether the cells are harvested fresh from culture or administered directly from cryopreservation influences their ability to secrete cytokines and interact with other cell types [63–65].

**Fig. 1:**
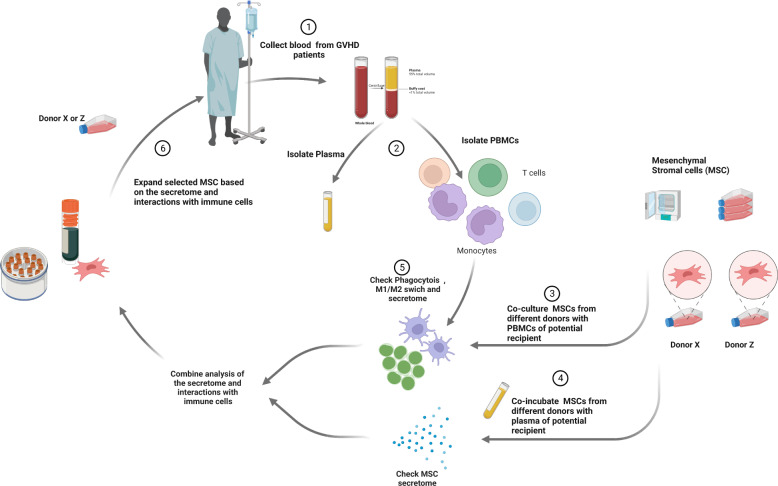
Personalized MSC therapy for GvHD. Blood from a potential MSC recipient with GvHD is collected (1). Peripheral blood mononuclear cells (PBMCs) and plasma from this patient are isolated (2). Mesenchymal stromal cells (MSCs) are generated from the bone marrow of different donors and incubated with PMBCs (3) and/or plasma (4). The secretome of both MSCs and immune cells, as well as functional alterations of monocytes and T cells, are analyzed after coincubation (5). Based on the immune-modulatory readout, the most suitable MSCs are selected, expanded and infused into the patient (6)

Several trials with 30 or more patients, both retrospective and interventional, have been published with varying outcomes (Table 1). Many factors differ between studies and are likely to have skewed the outcomes: expansion protocols, MSC dose per infusion, number of infusions, patient age (pediatric versus adult patients), and choice of second-line agents. The use of potency assays to quantify the viability and fitness of a cell batch may help clarify the large variability in patient outcome [66] (Fig. 1).

**Table 1:** MSC treatment studies with 25 participants or more

Reference, year	Cohort	Acute GvHD grade	Dose of MSCs, cells/kg body weight	Number of doses	Response rate Day + 28	Survival
Le Blanc et al. 2008 [37]	*n* = 55 25 children 30 adults	II: *n* = 5 III: *n* = 25 IV: *n* = 25	1.4 × 10^6^	1 (*n* = 27) 2 (*n* = 22) 3–5 (*n* = 6)	CR = 54.5% PR = 16% OR = 70.5%	2-year OS 35%
Resnick et al. 2013 [45]	*n* = 50 25 children 25 adults	II–III: *n* = 8 IV: *n* = 42	1.05 × 10^6^ (average first dose)	1–4	CR = 34% OR = 66%	3.6-year DFS 56%
Sánchez-Guijo et al. 2014 [72]	25 adults	II: *n* = 7 III: *n* = 13 IV: *n* = 3	1.1 × 10^6^	2 (*n* = 4) 3 (*n* = 3) 4 (*n* = 18)	CR = 44% PR = 27% OR = 71%	1-year OS 44%
Introna et al. 2014 [46]	*n* = 40 15 children 25 adults	II: *n* = 11 III–IV: *n* = 20 cGVHD: *n* = 3 overlap: *n* = 6	1.5 × 10^6^	3 (children 2–7) (adults 2–11)	CR = 27.5% PR = 40% OR = 67.5%	1-year OS 50% 2-year OS 38.6%
Zhao et al. 2015 [75]	*n* = 28 Age 14–54	II: *n* = 4 III: = 8 IV: = 16	1 × 10^6^	4 (2–8)	MSCs vs. ctrl OR: 75% vs. 42% CR: 60 vs. 26%	MSCs vs. ctrl 3 year OS: 46% vs. 26% (f/u 1.5–44 m)
Te Boome et al. 2015 [69]	*n* = 48 7 children 41 adults	II: *n* = 12 II: *n* = *33* III: *n* = 3	1.8 × 10^6^	1–4	CR = 25%	1-year OS 44%
von Dalowski et al. 2016 [68]	58 adults	I: *n* = 1 II: *n* = 3 III: *n* = 8 IV: *n* = 46	0.99 × 10^6^	1–2 (*n* = 40) >3 (*n* = 18)	CR = 9% PR = 38% OR = 47%	100-day OS 34.5% 2-year OS 16.6%
Servais et al. 2018 [70]	*n* = 33 4 children 29 adults	II: n = 9 III: *n* = 15 IV: *n* = 9	1-2×10^6^ *n* = 20 3-4×10^6^ *n* = 13	1 (*n* = 25) 2 (*n* = 8)	CR = 21.9% OR = 40%	1-year OS 18%
Fernández-Maqueda et al. 2017 [76]	33 adults	II: *n* = 17 III: *n* = 9 IV *n* = 7	1.06 × 10^6^	4 (1–16)	CR = 33% PR = 48% NR = 15%	1-year OS 79% in CR patients vs. 25% in PR/NR
Bader et al. 2018 [79]	*n* = 69 51 children 18 adults	II: *n* = 3 III: *n* = 25 IV: *n* = 41	1–2 × 10^6^	1–4	CR = 31.9% PR = 50.7% OR = 82.6%	6-month OS 71 ± 6%
Salmenniemi et al. 2017 [77]	*n* = 30 8 children 22 adults	II: *n* = 2 III: *n* = 14 IV: *n* = 10 cGVHD: *n* = 4	2.0 × 10^6^	Up to 6 doses	CR = 23% VGPR = 13% PR = 17% OR = 53%	6-month OS 54% 2-year OS 29%
Dotoli et al. 2017 [67]	*n* = 46 16 children 30 adults	III: *n* = 10 IV: *n* = 36	Cumulative dose 6.81 × 10^6^	3 (1–7)	CR = 6.5% PR = 43.5% OR = 50%	100-day OS 34.4% 2-year OS 17.4%
Galleu et al. 2019 [71]	*n* = 60 4 months – 68 years	I-II: *n* = 5 III-IV: *n* = 55	2.6 × 10^6^	1 (*n* = 34) 2 (*n* = 16) 3 (*n* = 6) 4 (*n* = 1)	NRe	OS 104 days (0-215)
Hinden L, et al. 2019 [88]	*N* = 26 Both <18 and >18	I-II: 3 responders and 2 non responders) III-IV: 10 responders and 11 non responders	0.59 to 1.8 million	1 (*n* = 26)	NRe	OS 40 days 11 (84.6%) responders and 4 (30.8%) non responders
Kebriaei P, et al. 2020 [55]	*N* = 163 with MSCs (M) and 81 controls without MSCs (C). 6 m to 70 years	II: 37 M, 21 C III: 82 M, 46 C IV: 44 M, 14 C	2 × 10^6^	8 (given over 4 weeks)+ 4 for IR group	DCR = 36.8% M versus 32.1% C OR = 58.3% M versus 54.3% C	180 days, M: 34% C: 42%
Kurtzberg J, et al. 2020 [132]	*N* = 54	II: *n* = 6 III: *n* = 23 IV: 26	2 × 10^6^	8 (given over 4 weeks	CR = 29.6% PR = 40.7% OR = 70.4%	180 days, 68.5%
Ke Zhao et al. 2022 [4]	*N* = 101 with MSCs (M) and 102 controls without MSCs (C) 14 to 65 years	II: 36 M, 37 C III: 41 M, 44 C IV: 22 M, 18 C	1 × 10^6^	4 doses (every week)	CR = 56.6% M versus 40.4% C PR = 26% M versus 30.3% C NR = 17.2%M versus 29.3% OR = 82.8% M versus 70.7% C	M: 11.3 months C: 6 months

*aGvHD* acute graft versus host disease, *cGvHD* chronic graft versus host disease, *MSCs* mesenchymal stromal cells, *OS* overall survival, *DFS* disease-free survival, *CR* complete response, *VGPR* very good partial response, *PR* partial response, *OR* overall response, *NRe* not reported; *M* MSC treatment arm, *C* control arm

Not all studies have indicated effectiveness. In a retrospective, multicenter study of three public hospitals in Brazil, response to healthy allogeneic, unrelated bone marrow MSCs in patients with SR-aGvHD could be detected in 23 out of 46 patients (50%), with only three patients (6.5%) obtaining CR [67]. A study by von Dalowski et al. showed similar results in adult patients (median age 55 years). The estimated 1-year OS was 19% and did not significantly differ from that of historical controls treated with the best available therapy [68]. A Dutch prospective multicenter phase II study using MSCs from BM aspirates obtained from third-party, HLA-disparate healthy donors expanded in medium with platelet lysate showed CR in 12 patients out of 48 (25%) at Day 28 [69]. The one-year OS was significantly improved in responding patients compared with nonresponding patients, as previously demonstrated [37, 44]. In another prospective multicenter study from Belgium, only five out of 33 patients receiving unrelated MSCs achieved sustained CR lasting at least one month, and the one-year survival was low (18.2%) [70]. Most of the patients in the cohort were over 50 years old. Interestingly, patients who received a higher MSC dose as their first infusion had higher response rates and survival than patients receiving a lower dose (3-4 versus 1-2 million MSCs/kg). In summary, several factors are associated with improved responsiveness to MSC therapy, including a high cell dose, younger patient age [37, 45, 46, 71], and gut and/or skin involvement [71–73]. However, these observations were not confirmed by others [74], and the mechanisms behind the differences in clinical response have not been fully deciphered.

A prospective, open-label, nonrandomized study from China compared 19 patients treated with allogeneic MSCs with 28 controls. The MSC-treated group had significantly higher CR rates and a trend toward improved OS. The 2-year cumulative incidence of cGvHD was 31.5% ± 10.1% in patients receiving MSCs compared to 79.2% ± 12.7% in controls (*p* = 0.045). In addition, the MSC-treated cohort was at significantly lower risk of developing extensive cGvHD (1/23 vs. 5/12, *p* = 0.005) [75].

Two Spanish trials produced additional encouraging results. In a multicenter phase II study, cryopreserved BM-derived MSCs expanded in platelet lysate were given to 25 patients as second-line treatment for grade II–IV SR-aGvHD. At least two doses were given to each patient, with 21 patients receiving 3 or more doses [72]. The CR rate was 42%, and the overall response rate was 71%. Those patients with skin and GI involvement had better outcomes, and no other clinical or laboratory parameters correlated with responsiveness to MSC therapy. Patients with CR had higher one-year survival.

The second study, a single-center trial from Madrid, used allogeneic MSCs isolated from third-party BM that were cultured, expanded, cryopreserved and infused directly after thawing [76]. Thirty-three patients were included with a median age of 46 years (18–61). Again, patients with CR by Day 28 had higher Day 90 survival rates (100% vs. 47.6%; *p* = 0.006) and increased OS over time.

Salmenniemi et al. used allogeneic third-party bone marrow-derived MSCs and reported the outcomes of 30 consecutive patients from Turku and Helsinki, Finland (22 adults and 8 children) [77]. The majority of patients (92%) had severe grade III-IV aGvHD [77]. At Day 28, the OR rate was 62%, which was higher among children than adults (50% and 88%, respectively). The survival rate of pediatric patients was significantly higher than that of adults (88% versus 22%, *p* = 0.003). Despite relatively high response rates, only 22% (4/18) of adults were still alive after the median follow-up of two years, primarily due to late infectious complications.

To prevent variability between MSC batches that may result in inconsistent results after treatment, Kuçi and colleagues developed an MSC bank made from a pool of BM mononuclear cells from eight healthy (HLA-disparate) donors. The cells were cultured in platelet lysate-supplemented media and frozen in aliquots that could be used for further expansion [78]. The MSC product, MSC-Frankfurt am Main (MSC-FFM), received national marketing authorization in Germany based on the “hospital exemption” clause of the European Advanced Therapy Medicinal Product (ATMP) guidelines. Although the hospital exemption clause allows the use of unapproved therapeutics only on a national level, treatment of the first 69 patients took place in 14 different transplant centers in six countries [79]. Most patients had aGvHD of grade III (36%) or IV (59%). A dose of 1-2 million MSCs/kg recipient weight once weekly for 1–4 weeks was the recommended treatment regimen. By Day 28, 83% of patients had responded, with CR in 22 (32%) and PR in 35 (51%) of the recipients. At the last follow-up (median 8 months, range 0.9–54 months), 61% of patients were in CR, 25% were in PR, and 14% were nonresponders. In contrast to several earlier reports, the ORs at the last follow-up after the first administration of MSCs were similar among children and adults, 89% and 84%, respectively. After receiving MSC-FFM treatment, the estimated OS at six months was 75% for patients with grade III and 67% for patients with grade IV aGvHD.

A retrospective analysis of a cohort of 60 SR-aGvHD patients treated with allogeneic BM-derived MSCs between 2008 and 2014 at several UK centers was published by Galleu and his coworkers in 2019 [71]. In contrast to other reports, the MSC treatment response was evaluated one week after administration rather than on Day 28. The aim was to identify an early predictor of clinical outcome. Thirty-four patients received one dose of MSCs, while 23 received multiple doses (2 to 4). Overall effectiveness of MSC infusion was detected in 32 patients (53%). With the exception of two patients, repeated infusions of MSCs did not alter the type of response obtained after the initial dose, agreeing with findings previously reported by Le Blanc et al. [37]. Overall survival was significantly affected by responsiveness to MSCs, with responding patients having significantly longer OS (*p* < 0.0001). In multivariate logistic regression analysis, age (younger than 20 years), higher MSC dose and gut involvement, skin involvement or both (compared to liver involvement) were prognostic factors for response. These findings are in agreement with results reported by Sevais et al. indicating that patients who received an MSC dose of more than 3 million/kg had better responses and longer survival than patients treated with an MSC dose of less than 3 million/kg [70].

As previously mentioned, a subset analysis of pediatric participants in the original remestemcel-L study suggested that pediatric patients treated with MSCs had a higher OR than patients given placebo (64% versus 23%; *p* = 0.05) [55]. In 2020, an update on pediatric patients treated within the expanded access program was reported by Kurtzberg et al. [80] The study included the 75 patients reported previously and encompassed a total of 242 children with severe SR-aGvHD from 50 locations in eight countries, with 232 completing the treatment protocol. The primary initial endpoint of CR on Day 28 used in the randomized phase III trial [55] was adjusted to include patients with PR in the expanded access program. This was done in accordance with estimated predictions of OS that were seen for patients in many of the academic studies that were conducted in Europe, following either a complete or partial response. The biweekly treatment regimen of 2 × 10^6^ MSCs/kg for 4 weeks was consistent throughout the various trials. Patients who achieved either PR or a mixed response were given an additional four weekly infusions after Day 28. The average age was 9.6 years (0.3 months to 18.2 years), and most patients had grade III (30%) or grade IV (50%) aGvHD. Before receiving remestemcel-L, 190 patients (78.8%) had undergone at least three or more nonsteroidal aGvHD treatments, indicating that the study population was severely ill and refractory to multiple therapies. A total of 156 patients (65.1%) met the primary endpoint of OR on Day 28, with 34 (14.1%) achieving CR and 123 (51.3%) achieving PR. Survival through Day 100 (a secondary endpoint) was 66.9% and was significantly higher in patients with OR on Day 28 than in nonresponders (82.1% vs. 38.6%; *P* < 0.001).

Based on the data described earlier, a phase III, prospective, single-arm, multicenter study in 54 children with primary SR-aGvHD who were naïve to other immunosuppressant therapies was set up following the same treatment protocol [80]. Based on published age- and disease severity-adjusted findings, as well as internal data [81], the study outcome was compared to a predefined null hypothesis of 45% OR for standard of care alone. When compared to the predetermined OR rate, the OR on Day 28 of remestemcel-L therapy was significantly increased (70.4% compared with from 45%; *p* = 0.0003). The higher OR (70.4%) was sustained through Day 100 and included an increase in CR from 29.5% on Day 28 to 44.4% on Day 100. At Day 28, the OR in the study subjects was highly predictive of improved 180-day survival. The researchers concluded that remestemcel-L treatment was a safe, tolerable, and efficient treatment for SR-aGvHD in pediatric patients.

Based on the available evidence, Mesoblast submitted a Biologics License Application (BLA) in the spring of 2020 to treat children with SR-aGvHD with remestemcel-L. To provide an impartial and independent estimate of response rates and outcomes, the clinical submission included an analysis of 309 children with GvHD who received remestemcel-L. Response to MSC infusion was compared with data on 30 matched pediatric control patients from the MAGIC consortium’s database. Although the FDA Advisory Committee voted nine to one in favor of remestemcel-L (now known as RYONCIL^TM^), the application was declined, and MSC treatment is not approved as a therapy for GvHD in the US.

Earlier this year, Zhao Ke et al. reported a phase III multicenter randomized controlled trial involving 203 SR-aGvHD patients [4]. Patients were treated with basiliximab and a calcineurin inhibitor with (*n* = 101) or without (*n* = 102) MSCs. The addition of MSC treatment improved OR at Day 28, durable response at Day 56, and median failure-free survival and reduced the 2-year cumulative incidence of cGvHD development. The 3-year cumulative risk of leukemia relapse was similar in both groups. One important finding from this study was that MSCs reduced the side effects of basiliximab and calcineurin inhibitors, such as bone marrow toxicity, suggesting that MSCs support the hematopoietic niche. Infections were also reduced in the MSC group; this may reflect improved immune reconstitution by MSCs. Although basiliximab would be an unusual choice of second-line treatment for SR-aGvHD in parts of the world, the study was importantly the first with a specified-second line therapy design. In addition, the authors stated that when nonresponding patients were switched to ruxolitinib, patients in the MSC arm displayed higher responses than the control group (42.8% MSCs group vs. 11.1% control), although the total number of such patients was too small for conclusions to be dawn (7 in the MSC group and 9 controls).

## Biomarker studies on MSC products and aGvHD patients–understanding the mode of action

Due to the multitude of available second-line therapies for GvHD and the heterogeneity of the disease, identifying biomarkers that differentiate MSC responders from nonresponders is important. The MSC field initially focused on identifying markers that could reliably predict the clinical efficacy of a specific MSC batch in a particular group of patients. To date, a reliable potency marker is still lacking. To better understand the function and efficacy of MSCs, the International Society for Cellular Therapy has recommended a series of functional tests, including quantitative RNA analysis of selected gene products, either from resting MSCs or from MSCs that have first been licensed with IFNγ, a protein-based assay of the secretome and flow cytometry analysis of functionally relevant surface markers [82]. However, the response to MSC infusion may be disease-specific. For example, infused IFNγ-prelicensed MSCs effectively protected mice against lethal acute radiation syndrome but failed to protect mice with GvHD [83]. In vitro, in coculture experiments with remestemcel-L, the FDA accepted MSC expression of tumor necrosis factor receptor type I (TNFR1) at a threshold level and inhibition of mononuclear cell expression of interleukin 2 (IL2) receptor α (CD25) as markers of potency and activity. However, these markers have failed to predict patient responsiveness and may rather be functional characteristics of cells with established MSC morphology and phenotype [74, 82].

Early work failed to correlate MSC suppression of lymphocyte proliferation in vitro with the MSC ability to alleviate GvHD [61].

In vivo, MSCs are tissue-resident cells and lack the inherent anticoagulant properties that make endothelial cells compatible with blood [84]. Instead, when MSCs are infused intravenously, adhesion receptors and surface matrix proteins (and in the case of adipose and placental-derived MSCs, also tissue factor expression) activate the complement and coagulation cascades, initiating an immediate blood-mediated inflammatory response (also known as IBMIR) leading to cell graft destruction [84]. Despite this “hit-and-run” fate of MSCs, several trials have indicated that MSC treatment increases T regulatory (Treg) cells compared to T helper (Th)1 and Th17 cells in responding patients but not in nonresponders [85, 86]. Te Boome et al., on the other hand, did not detect changes in Treg, CD4^+^ or CD8^+^ cell levels in any patients but observed a significant increase in immature dendritic cells in MSC responders [69]. Similarly, Keto et al. could not link changes in T-cell subsets with response but showed that lymphocyte levels were very low overall, even below the detection limit in many patients [87]. It can be assumed that for MSCs to have an immunomodulatory effect, the lymphocyte compartment of the recipient must be sufficiently large to skew toward a more tolerogenic profile. This notion is supported by an observation made by Hinden et al., who found the number of lymphocytes, especially NK and T cells, to be higher in responders than in nonresponders when measured before administration of MSCs [88].

Galleu et al., using a murine GvHD model as well as patient samples, demonstrated that infused MSCs are actively induced to undergo apoptosis by recipient cytotoxic cells after infusion [89]. The apoptotic MSCs are subsequently engulfed by phagocytes that in turn become licensed to produce indoleamine 2,3-diosygenase (IDO), a protein associated with MSC-induced immunosuppression. These results support the findings of earlier experimental models suggesting that phagocytes are mediators of MSC-induced adaptive responses [90–92].

A postulated MoA involving MSC destruction, efferocytosis and monocyte skewing agrees with the generally accepted dogma that HLA matching between MSC donors and recipients is not required for treatment efficacy, although the correlation between MHC expression and in vivo immunogenicity has rarely been studied [93–98].

According to Galleu et al., MSC cytotoxicity is mediated by CD8^+^ T cells and NK cells and is MHC independent [89]. Patients displaying high cytotoxicity respond to MSC therapy, while patients with low or absent cytotoxic activity do not experience disease improvement. Differences in donor cytotoxic responses may explain why, in a large number of clinical trials, responses have varied between patients who were treated with MSCs from the same donors [37, 42, 44, 47, 57, 68, 70].

We observed how important the immune repertoire in the GvH-affected organ can be for MSC responsiveness. The inflammatory profile of gut biopsies obtained from patients with GI GvHD prior to MSC infusion differed significantly between responding and nonresponding patients [99]. At the time of GvHD diagnosis, the gut mucosa of patients who later responded to MSCs had increased mast cell activity, CD8^+^ T cells, and Forkhead Box P3 (FoxP3)^+^ cells and lower levels of CD4^+^, CD56^+^ and CD68^+^ cells compared to that of patients who did not respond to MSCs. Thus, both CD4^+^ and CD8^+^ Treg cells may be important mediators of the MSC response.

REG3α, ST2 and other suggested markers of gastrointestinal crypt damage have been reported by the MAGIC consortium to correlate with long-term outcome, but their role in predicting responsiveness to MSCs is less clear. Conflicting results have been reported for some soluble disease markers. Dander et al. found that MSC treatment responders had lower plasma levels of elafin, IL-2R, and TNFRI [86]. Yin et al. showed that REG3α and CK18, an intermediate filament protein that indicates damage to the liver, were decreased in MSC responders [100]. In their phase I study, Introna et al. found significantly lower plasma levels of IL2R α (sCD25) in responders (CR and PR) than in nonresponding patients [46]. A study by Keto et al. showed that patient samples had significantly higher serum concentrations of REG3α, CK18F, and elafin than samples from healthy controls; however, only CK18 (a tissue damage marker for the liver and intestine) had the potential to predict MSC responsiveness [87]. Te Boome et al. found that ST2 was not predictive of therapy resistance before infusion of MSCs, as previously suggested [69]. However, two weeks after the first infusion of MSCs, a continued high level of soluble ST2 correlated with an increased risk of death.

An in-depth biomarker analysis of a subgroup of the pediatric cohort treated with remestemcel-L [80] indicated that children with biologically high-risk SR-aGvHD do benefit from remestemcel-L therapy. The survival of children receiving MSCs for high-risk disease, as defined by the MAGIC criteria, was significantly higher than that in a similar high-risk patient cohort from the MAGIC database treated with the best available therapy (64 vs. 10%).

## Mesenchymal stromal cells in chronic GvHD

Compared to aGvHD, fewer studies have evaluated the efficacy of MSCs for the prevention and treatment of cGvHD. Thymic damage causing dysregulation of adaptive and innate immune cells results in an immunocompromised status in which patients are at risk of infection and secondary malignancy. First-line treatment with glucocorticoids and calcineurin inhibitors will further increase the patients’ immune incompetence. It could be hypothesized that it is in this setting that MSCs are particularly useful because they provide both anti-inflammatory signals and trophic effects that aid restoration of a dysfunctional immune compartment. While the majority of trials where MSCs were used to treat cGvHD have been small and the data are therefore hard to reconcile, the latest available meta-analysis described promising response rates in two-thirds of refractory cGvHD patients after MSC infusion [101]. The studies differed in the tissue source of MSCs, number of infusions and immunological status of the recipients. We believe that the importance of composition and fitness of the immune system of the host before MSC infusion is underestimated, and these factors should be assessed when selecting patients for MSC treatment (Fig. 1).

### B cells in cGvHD: the unusual suspects

In 2010, Weng J et al. published the first promising MSC clinical trial on cGvHD with responsiveness in more than 70% of 19 infused patients [102]. The authors showed that CD8^+^ CD28^+^ T cells decreased and CD19^+^ CD5^+^ B cells increased as cGvHD improved. A separate study suggested an increase in regulatory CD5^+^ B cells after MSC infusion in cGvHD patients [103]. This study showed a CR or PR in 78% (20 out of 23) of patients and a significant increase in IL-10-producing B cells.

In a prophylaxis study, Gao et al. showed that the number of memory CD27^+^ B cells increased with MSC treatment [104]. Interestingly, patients who later developed cGvHD were deficient in memory CD27^+^ B lymphocytes [105]. Therefore, the increased CD27^+^ B cells after MSC treatment might represent a regulatory B-cell pool, as described by others [106], but this needs further investigation.

Recently, our group conducted a phase II clinical trial in severe refractory cGvHD patients treated with up to nine monthly MSC infusions [107]. The infusions were well tolerated. Six patients responded to MSC treatment according to the National Institutes of Health response criteria, accompanied by improvement in GvHD-related symptoms and quality of life. This response was durable, with systemic immunosuppressive therapy withdrawn from two responders, and two additional patients were able to discontinue steroid treatment and undergo calcineurin inhibitor tapering.

After each treatment, we observed an increase in the naïve B-cell population, but the characteristics of the memory B-cell population were unchanged [107]. Although these data are interesting, the available studies are too limited to draw firm conclusions regarding the role of B-cell subsets after MSC infusion for cGvHD.

### T cells in cGvHD: the usual suspects

In our study, not only B-cell frequencies but also naïve T-cell frequencies (with a high proportion of newly emigrant CD31^+^ cells) were elevated prior to treatment in the responders [107]. This suggests that thymic function plays a role in the responsiveness to MSCs, emphasizing the importance of investigating the host immune system prior to treatment. We believe that the immunomodulatory properties of MSCs depend not only on the donor but also on the microenvironment of the host [3].

In the phase III trial of aGvHD treatment by Zhao et al., which included more than 200 patients, the cumulative incidence of cGvHD was lower in MSC recipients than in the control group (39,5% vs. 62,7%) 2 years after infusion [4]. The authors suggested that MSCs play an important role in decreasing the severity of aGvHD-mediated thymic damage by decreasing autoreactive T cells and/or inducing Treg production [4]. Furthermore, Hinden et al. treated 26 patients with SR-GvHD (4 chronic and 22 acute) after allogeneic HSCT with MSCs and showed that the number of T cells was increased in responders compared to nonresponders [88]. In contrast, in a study by Gao et al., the total number of T cells did not change after MSC infusion, but Treg levels and the Th1:Th2 cell ratio increased [104]. These findings suggest that MSCs not only regulate T-cell homeostasis but also restore the balance between Th1 and Th2 cells. Results reported by Dander et al. were similar; the number and function of CD4^+^ and CD8^+^ T cells remained unchanged, but the proportion of Tregs compared with Th1 and Th17 cells was increased in MSC responders [86].

### Soluble factors in cGvHD: the good, the bad and the ugly

CXCL9 and CXCL10 are IFN-inducible chemokines that bind to the chemokine receptor CXCR3, their only known receptor, expressed by activated T cells [108]. Binding to CXCR3 promotes the recruitment of alloreactive T cells in cGvHD and likely drives pathogenesis [109]. Boberg et al. found that the levels of CXCL9 and CXCL10 (probably secreted by monocytes, macrophages and endothelial cells) predict responsiveness to MSC therapy for cGvHD. Both chemokines were increased in nonresponders early during treatment but remained stable in responders [107]. Biomarkers that can indicate responsiveness to MSCs (or any other therapy) are of particular importance in cGvHD patients, in whom the clinical response occurs slowly.

The efficacy of MSCs in cGvHD varies from study to study, possibly as a result of both donor and recipient heterogeneity [101]. How infused MSCs interact with the immune cells in cGvHD remains to be fully elucidated. While the focus on the past has mostly been on MSC interactions with T and B cells, the role of innate immune cells (e.g., monocytes) in cGvHD after MSC infusion has been investigated to a lesser extent. In 2018, Takaaki Konuma et al. reported monocyte alterations in progressive cGvHD [110]. It is well known from in vitro and in vivo studies that engulfment of MSCs skews monocytes and macrophages toward an anti-inflammatory and regenerative phenotype [89, 92, 111, 112]. Progress will require improved understanding of MSC function and the development of potency assays that help optimize donor-recipient matching to enhance responsiveness.

## MSCs in mouse models of GvHD

MSCs improve GvHD in some but not all experimental animal models. Tobin et al. studied the ability of human bone marrow MSCs to alleviate GvHD [113]. The authors used a humanized mouse model of aGvHD based on delivery of human peripheral blood mononuclear cells (PBMCs) to nonobese diabetic (NOD)-severe combined immunodeficient (SCID) interleukin (IL)-2rγ-null (NSG) mice. While liver and gut GvHD improved after MSC treatment and survival increased, MSCs failed to prevent GvHD development. MSC infusion did not induce the generation of regulatory T cells, PBMC apoptosis or T-cell anergy, suggestive of immune tolerance. Improvement was rather mediated by direct inhibition of donor CD4^+^ T-cell proliferation and decreased serum levels of TNFα. Similarly, in a xenogenic aGvHD model in which sublethally irradiated NOD/SCID mice were transplanted with human PBMCs, a single dose of umbilical cord-derived MSCs did not prevent disease, while weekly doses decreased T-cell proliferation and rescued mice from GvHD [114]. However, once established, MSCs failed to improve GvHD.

In contrast, bone marrow MSCs were not effective in preventing GvHD even when multiple doses were administered in two humanized aGvHD mouse models (NOD/SCID and NSG mice transplanted with PBMCs) [115, 116]. To address the effect of tissue origin on GvHD outcome, Grégoire et al. reported that administration of MSCs from both bone marrow and umbilical cord slightly prolonged overall survival in a model of xenogenic GVHD mice, suggesting that both cell products were effective [62]. Interestingly, adipose-derived MSCs were associated with coagulopathy and sudden death.

Nevertheless, a recent study suggested a beneficial effect of MSCs [117]. GvHD was established by transplanting C57BL/6 donor bone marrow cells and C57BL/6 EGFP (enhanced green fluorescent protein) splenocytes into lethally irradiated BALB/c nude recipient mice. MSCs accumulated in spleens, but not in lymph nodes, of mice transplanted with allogeneic hematopoietic cells but not in the group receiving syngeneic hematopoietic cells, suggesting that MSCs have tropism for active inflammation. However, MSCs do not exclusively migrate to the spleen in GvHD. Earlier studies suggested that intravenously infused MSCs were first detected in the lungs and then migrated to the gastrointestinal tract, lymph nodes and skin [118].

Regardless of the migration status, how MSCs prevent aGvHD in murine models remains unclear. Vacaru et al. proposed a mode of action that involves the FasL pathway and showed that treatment with MSCs that overexpress FasL resulted in delayed GvHD onset and increased survival [119]. In another study, Wang Rui et al. reported that MSCs derived from the umbilical cord express high levels of CXCL1, leading to the accumulation of myeloid-derived suppressor cells that control GvHD [120]. This leads to the question of whether the MSC effect is local and requires migration to the site of inflammation. In an elegant study, Court et al. showed that bone marrow MSCs can transfer mitochondria to CD4^+^ T cells [121]. In a murine aGvHD model, transplantation of human T cells boosted with artificially transferred MSC mitochondria significantly improved survival and reduced tissue damage. These data are in line with studies showing that MSCs transfer mitochondria to monocytes and other cells in vitro [122, 123]. Thus, in addition to their ability to differentiate and produce trophic and immunomodulatory factors and extracellular vesicles, MSC mitochondrial transfer might represent yet another MSC MoA. However, the factors that trigger MSC mitochondrial transfer have not been fully clarified.

Inflammatory signals from the surrounding milieu might play an important role in the therapeutic effects of MSCs in aGvHD. Galleu et al. transplanted lethally irradiated C57BL/6 male mice with polyclonal purified CD4^+^ T cells from female syngeneic donors and purified CD8 + T cells transgenic for a T-cell receptor specific for the male mouse HY antigen [89]. In this setting, bone marrow MSCs were actively induced to undergo perforin-dependent apoptosis by both recipient cytotoxic and phagocytic cells, a process deemed essential for the therapeutic effects of MSCs. Such data are consistent with our previous findings of rapid engulfment of MSCs by monocytes after their opsonization with complement factors [90]. Thus, the inflammatory milieu in GvHD might induce MSC efficacy. This agrees with findings suggesting that cyclosporin A treatment reduces MSC suppression. The effect can be overcome by priming MSCs with IFNγ for 24 h before exposure to cyclosporin A, enhancing the immunomodulatory capacity of MSCs both in vitro and in a humanized mouse model of aGvHD [124]. However, IFNγ might not be solely responsible for the enhanced potency of MSCs, as another group reported that infusion of IFNγ-licensed allogeneic MSCs failed to mitigate acute GvHD in another murine model [83].

Although existing mouse models reflect human GvHD biology to some extent, species-specific factors characteristics, including important mediators of MSC-induced immune suppression, differ between humans and mice, suggesting the need for more relevant models.

## Consolidation of data towards translation

A survey conducted by Trento et al. in 17 European academic centers reported the outcomes of MSC production and treatment of more than 1000 patients [59]. According to worldwide academic and commercial studies, the safety profile of BM-derived MSCs appears to be excellent, with no severe side effects reported. In a meta-analysis published in 2021, Wang Yang et al. included more than 3400 patients treated with i.v. or local MSC injections for different diseases [125]. The authors found no reports of serious safety events other than transient fever, insomnia, and constipation. Placenta- and umbilical cord-derived MSCs were used in only a few studies. MSCs sourced from tissues other than BM may have the same morphology, but they are metabolically different and have different procoagulant properties. Therefore, independent assessments of safety profiles are required when working with these cells [126, 127].

Most studies report that response to MSCs is associated with improved outcome [50]. Thielen et al. attempted to develop treatment algorithms using information from 14 phase II trials that covered 327 patients with grade II–IV aGvHD [128]. The probability of achieving CR within the first 28 days was 43.4%, and the median survival time for patients with CR was 3.2 years, compared to 6 months for incomplete responders. Understanding the biological parameters associated with treatment response has been more challenging. In general, better responses have been reported in pediatric patients than in adults.

The difficulty of designing late-phase trials that take into consideration the myriad factors that can introduce heterogeneity, such as differences in cell products, procurement, and particularly host factors [129, 130], is highlighted by the failure of Osiris’ phase III trial. The study design, which allowed for a variety of second-line therapies across sites and operational deviations from the protocol, likely obscured the trial’s outcome.

Placebo-controlled trials can be deemed unethical or impractical in lethal orphan diseases where there is no approved treatment, particularly if safety of the intervention has already been established in previous phase I/II studies. Recognizing these limitations, regulatory agencies in Japan, Europe, and the US have expanded on their initial regulatory frameworks in recent years to implement new, more adaptable, and speedier review procedures for cell and gene therapy products [131]. Temcell was granted approval in Japan as a result of these more adaptable regulations.

Despite the paucity of placebo-controlled trials, it is abundantly clear that MSCs are beneficial to individual patients. The expanded-access case series on pediatric patients treated with an identical MSC product and dosing scheme is an important example of this. The data compiled on hundreds of children treated with remestemcel-L provide important insights into the importance of empirical clinical observations.

## Summary

Multiple studies conducted around the world have shown an exceptional safety profile of MSCs and indicated their efficacy in treating GvHD. However, an assay that quantifies the in vivo immunosuppressive action of MSCs has remained elusive.

Clearly, clinical responsiveness depends on the interactions between the MSC product and host immune cells that take place in the inflammatory milieu of the recipient (Fig. 1). Implementation of late-stage trials with predefined clinical criteria has been difficult and has raised ethical concerns for patients with a lethal disease. In addition, such studies risk categorizing patients with similar clinical symptoms but vastly different underlying biology into the same category, a possible explanation for the diverse reported outcomes. It could be speculated that patients with severe lymphodepletion due to GvHD have lost responsiveness to MSCs. An exhausted lymphocyte pool could potentially result from not only immune reactions characteristic of a particular patient’s GvHD but also multiple immunosuppressive treatment regimens prior to MSC treatment. We recommend continued immune profiling of patients both before and after MSC treatment to identify the patients who, as observed by many investigators, will clearly benefit from MSC treatment of GvHD.
